# FunnyBase: a systems level functional annotation of *Fundulus *ESTs for the analysis of gene expression

**DOI:** 10.1186/1471-2164-5-96

**Published:** 2004-12-20

**Authors:** Justin E Paschall, Marjorie F Oleksiak, Jeffrey D VanWye, Jennifer L Roach, J Andrew Whitehead, Gerald J Wyckoff, Kevin J Kolell, Douglas L Crawford

**Affiliations:** 1 Division of Molecular Biology and Biochemistry, 5100 Rockhill Rd., University of Missouri-Kansas City 64110, USA; 2 Department of Environmental & Molecular Toxicology, North Carolina State University; Raleigh, NC 27695-7633 USA; 3 Division of Marine Biology and Fisheries, NIEHS Marine and Freshwater Biomedical Sciences Center, Rosenstiel School of Marine & Atmospheric Science, University of Miami, Miami, FL 33149, USA

## Abstract

**Background:**

While studies of non-model organisms are critical for many research areas, such as evolution, development, and environmental biology, they present particular challenges for both experimental and computational genomic level research. Resources such as mass-produced microarrays and the computational tools linking these data to functional annotation at the system and pathway level are rarely available for non-model species. This type of "systems-level" analysis is critical to the understanding of patterns of gene expression that underlie biological processes.

**Results:**

We describe a bioinformatics pipeline known as *FunnyBase *that has been used to store, annotate, and analyze 40,363 expressed sequence tags (ESTs) from the heart and liver of the fish, *Fundulus heteroclitus*. Primary annotations based on sequence similarity are linked to networks of systematic annotation in Gene Ontology (GO) and the Kyoto Encyclopedia of Genes and Genomes (KEGG) and can be queried and computationally utilized in downstream analyses. Steps are taken to ensure that the annotation is self-consistent and that the structure of GO is used to identify higher level functions that may not be annotated directly. An integrated framework for cDNA library production, sequencing, quality control, expression data generation, and systems-level analysis is presented and utilized. In a case study, a set of genes, that had statistically significant regression between gene expression levels and environmental temperature along the Atlantic Coast, shows a statistically significant (P < 0.001) enrichment in genes associated with amine metabolism.

**Conclusion:**

The methods described have application for functional genomics studies, particularly among non-model organisms. The web interface for *FunnyBase *can be accessed at . Data and source code are available by request at jpaschall@bioinfobase.umkc.edu.

## Background

Investigating patterns of gene expression using mouse and human microarrays has produced insights into cancer [1,2], cardiac diseases [3-6], and metabolic disorders [7-12]. These and many other functional genomics studies rely on full genomic sequence to establish well-annotated databases. Yet, microarrays based on EST collections are increasingly being used for diverse species, from honey bees to fish [13-20] and including simple diploblastic organisms [21]. These studies within a diversity of organisms provide insights not provided by 'model' species (species that are genetically well defined or with annotated genomes [22]). For example, 'non-model' organisms have provided insight into the natural variation in gene expression [23], social castes among bees [24,25], hypoxia [26], and physiological responses to variation in the thermal environment [27,28]. To investigate adaptive variation in gene expression we use the teleost *Fundulus heteroclitus *(killifish) [23,29].

The killifish *Fundulus heteroclitus *are distributed along the eastern coast of North America which has one of the steepest thermal clines in the world: northern populations have environmental temperatures more than 12°C below southern populations across 12 degrees of latitude. Migration among populations is sufficient to minimize random genetic drift [30] but not frequent enough to extinguish local adaptation [31,32]. Populations are large (>10,000) and affected by historical, demographic and selective constraints, providing a framework for the partitioning of variation in gene expression within and among populations. Additionally, the well-established phylogenetic relationship among *Fundulus *species can be used to discern adaptive changes [23,33,34]. These characteristics make *F. heteroclitus *an ideal species to investigate adaptive variation in gene expression.

Microarrays from diverse EST collections offer opportunities to address many biological problems, but to effectively use this information often requires a locally generated bioinformatics approach. Tools like the TIGR Gene index [35] and Unigene [36] provide significant information on many species, yet these databases do not meet the needs of functional genomics projects for many non-model species. Currently, TIGR and NCBI provide gene indices for 28 and 23 animal species, respectively. Yet, there are 63 animal species with more than 10,000 ESTs [37]. The number of species with ESTs >10,000 has continued to grow, and there was approximately a 20% increase in the preceding three months. While annotation from these resources can be accessed through web-based homology searches, for many laboratory collections of ESTs it is difficult to use existing tools to achieve a systems-level view of gene functions and relationships. Rather than simply browsing functional information over the web for a different group's project, laboratories that produce novel EST collections and microarrays require customized databases providing access to integrated functional annotation as expression data are being analyzed.

We have developed *FunnyBase *to meet these functional genomics needs. *FunnyBase *provides functional information for >40,000 ESTs from the teleost fish *Fundulus heteroclitus*, provides the means to quickly process, evaluate, and store annotation based on similarity searches of public resources, and integrates these data with species-specific clustering and microarray analysis. Perhaps ironically, the greatest challenge for functional annotation based on similarity searches is an overabundance of data. There are a number of databases to chose from, and often the single best hit from a given database search is not the most informative. *FunnyBase *implements a strategy to make maximum use of systems-level functional information from Gene Ontology (GO) [38] assignments and membership in metabolic pathways as defined by the Kyoto Encyclopedia of Genes and Genomes (KEGG) [39]. Specifically, several sequence databases are queried and results integrated to maximize the number of annotated sequences. Alignments and scores for all homology based associations are tracked, allowing further evaluation and statistical studies.

Microarray data using genes annotated in *FunnyBase *can be systematically analyzed in the context of biological functions. We present a case study to illustrate how assessment of systems-level annotation can identify statistically significant functional differences among sets of genes.

## Results and discussion

**Figure 1:**
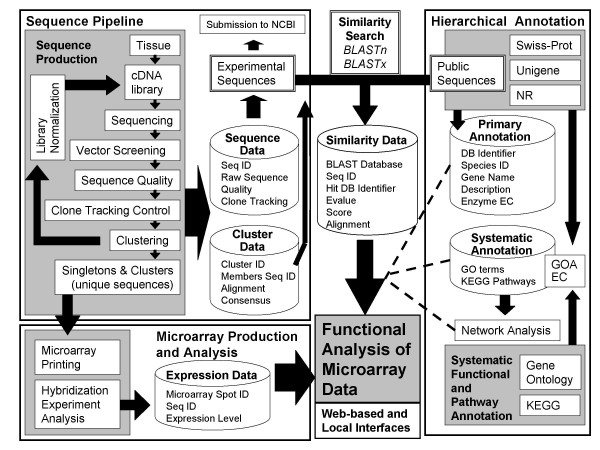
*FunnyBase *annotation scheme. The integration of the three *FunnyBase *modules: sequence pipeline, hierarchical annotations and microarray production and analysis. Database tables are shown in cylinders, arrows are data flow, and dashed lines indicate the integration of data from multiple sources.

*FunnyBase *(Fig. 1) is divided into 3 modules: Sequence Pipeline, Hierarchical Annotation, and Microarray Production and Analysis. The Sequence Pipeline takes sequences and quality output files from the sequencer, applies vector screening, quality trimming, clone tracking, and clustering (described below) to produce a set of unique sequences that are deposited in the 'Sequence Data' and 'Cluster Data' tables. Notice "unique sequences" are a combination of singletons (single unique ESTs) and clusters of overlapping sequences.

The Hierarchical Annotation module uses the consensus sequences from the clusters or singletons, and integrates primary annotation such as gene name and description with associated pathways and systems-level functional annotation. This may include gene function (e.g., enzyme catalyst), metabolic or signal pathway (e.g., oxidative phosphorylation), or biological function (e.g., protein translation). Sequence data from the first module and functional annotation from the second are matched using database similarity searches (BLASTX and BLASTN). E-values, bit scores and local alignments are stored in the 'Similarity Data' table for all significant matches. One of the strengths of *FunnyBase *is the use of different sequence databases (SwissProt [40], NCBI UniGene [36], and NCBI non-redundant NR [41]) to provide separate annotations. Although these databases are not completely independent, the three separate annotations provide verification of gene names.

The third module, Microarray Production & Analysis provides a list of unique genes to be printed and integrates expression data from microarray experiments with the Hierarchical Annotation module. This provides functional annotation for expression data. *FunnyBase *annotation is accessible through the web or through local SQL queries and data-mining scripts.

### EST isolation and sequencing

The overall strategy used to isolate and sequence thousands of *Fundulus *cDNAs was (1) generate a high quality unidirectional cDNA library, (2) normalize the library, (3) randomly pick colonies and amplify by PCR the cDNA within the vector, (4) sequence and identify PCR products, and (5) after approximately every 1,000 clones, subtract these from the normalized library and repeat steps 3–5. Details for all protocols are provided at  and were used in the Comparative Functional Genomic course at Mount Desert Island, ME 2000.

We sequenced 46,433 ESTs and 40,043 of these are available in the dbEST database at NCBI (dbEST identification numbers: 23,480,307 to 23,515,306; 23,520,047 to 23,525,409 and 24,320,128 to 24,320,184) as of June 26, 2004. Sequences in *FunnyBase *are identified by a number series: unique sequence number, array number, plate number and well identifier (example: 23434_125_001_H04). The remaining 6,966 un-submitted sequences failed to meet one of the sequence quality parameters. Two criteria are used for defining "good" sequences: 1) >100 bp of sequence with Phred score >20 or 2) form an overlapping cluster with other sequences. Of the 19,937 sequences processed with the current version of *FunnyBase*, 17,893 (90%) passed one of these quality parameters. In earlier iterations of *FunnyBase*, visual inspection and later a sequencer-specific quality measure equivalent to a Phred score of 15 were used as filters resulting in 3,603 of the first 5,760 sequences (63%) and 13,922 of the next 15,168 sequences (92%) meeting quality standards, respectively. Re-sequences account for 5,668 sequences, and 4,625 of these were submitted.

### Controls

One of the most important steps for producing microarrays from cDNA libraries is being able to associate the bacteria containing the cDNAs of interest with the EST annotation. High-throughput procedures are highly prone to tracking errors including: loading plates into an automatic sequencer in the wrong order, orienting symmetric plates in the wrong direction, or mislabeling of plates. The ability to identify these types of mistakes requires controls for identifying plates and plate orientation. The *FunnyBase *system has a number of integrated quality control steps. First, a *Ctenophore *cDNA (NCBI: accession number: CN992733) that is unlike anything else in GenBank is used as a control. Controls are placed in 96-well plates in wells corresponding to the plate number and two orientation wells (A5 and F9). Sequences from 96-well plates are automatically scanned for these controls so that the identity and orientation are confirmed and a report is generated for manual review. Secondly, all clones used for microarray production are re-sequenced. This is necessary because individual cDNAs are cherry picked, re-grown and re-amplified, and each of these steps has the potential to introduce or magnify an error. For example, for a 6,000 gene array, a 5% error rate would result in 300 incorrect clones. Re-sequenced array plates are compared using pair-wise BLAST [42] against previous sequencing results so that the identity of printed microarray spots are verified.

### EST clustering

EST projects generate a number of redundant sequences due to the random selection of cDNAs from tissue libraries (Table 1). Clustering redundant sequences is a critical first step of analysis in order to identify genes to target for subtraction. The program CAP3 by Xiaong Huang [43] was used to cluster EST sequences with a 30 bp overlap and 75 percent similarity.

**Table 1:** The Ten Most Frequently Annotated ESTs. Clusters with the greatest number of annotated ESTs, the sequence id, number of ESTs that cluster together, and the e-value (probability of similarity) are listed.

**ID**	**Number of ESTs**	**Evalue**	**Description**
1616	977	0	Vitellogenin I precursor
2262	734	2e-88	Cytochrome c oxidase polypeptide II
1348	507	1e-102	Alpha-1-antitrypsin homolog precursor
1026	481	5e-61	Zona pellucida sperm-binding protein 3 precursor
1727	401	0	Serotransferrin precursor
555	397	0	Cytochrome c oxidase polypeptide I
640	369	8e-61	Zona pellucida sperm-binding protein B precursor
1549	351	2e-77	Apolipoprotein A-I precursor
570	331	1e-111	Cytochrome c oxidase polypeptide III
1178	304	2e-45	ATP synthase a chain

*FunnyBase *contains a total of 40,043 EST sequences from *F. heteroclitus *heart and liver. Clustering with CAP3 yields 3,776 clusters that contain 30,688 ESTs (77%). The remaining 8,991 ESTs (23%) are singletons. By storing the results of clustering with annotation, *FunnyBase *easily identifies these genes and aids in the selection of genes to be used for library subtraction with the goal of picking less common transcripts.

The 10 annotated clusters with the most sequences are listed in Table 1. In microarray experiments these genes tend to be highly expressed and the fluorescent signal tends to saturate the photomultiplier tube. These genes also serve to verify microarray printing because the predicted spots for these genes have the strongest signal.

**Figure 2:**
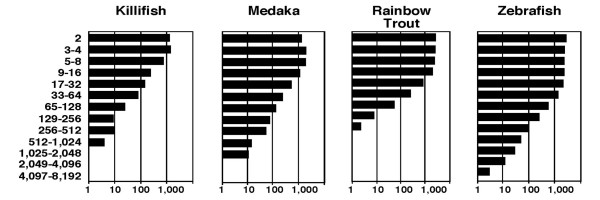
Frequency of cluster size class in teleosts. The frequency of the number of ESTs in each cluster is shown for *Fundulus heteroclitus *(Killifish: total number of clusters 3,779), *Oryzias latipes *(Medaka: total number of clusters 7,401), *Oncorhynchus mykiss *(Rainbow Trout: total number of clusters 11,405) and *Danio rerio *(Zebrafish: total number of clusters 14,714). For example, the first bar indicates that approximately 1,000 clusters contain 2 ESTs in all four teleost fish. Clusters for other species (not *F. heteroclitus*) are based on NCBI UniGene.

The distribution of the number of clusters with two or more ESTs is depicted in Figure 2. Although most clusters (2,581 or 68%) in *FunnyBase *have two or three-to-four sequences (Fig. 2), a small number of highly expressed genes form clusters with a large number of ESTs. For example, there are three clusters that contain 512 to 1,024 ESTs and ten clusters that contain 256 to 512 ESTs (bottom two bars for killifish in Fig. 2). This distribution is similar to other teleost fish EST collections (Fig 2). Notice, as more ESTs are added, clusters tend to get larger (more ESTs per cluster) rather than new small clusters growing in frequency. Of the 3,779 killifish clusters, 14% have more than eight ESTs, yet of the 14,714 Medaka clusters, 48% have eight or more ESTs. These distributions suggest that adding more EST sequences has diminishing returns.

One of the objectives of EST projects is to isolate most, if not all, genes expressed in a tissue or organism. The increasing size of larger clusters with more sequencing efforts indicates that strategies to increase the probability of isolating new genes need to be employed. We used two strategies. First, we normalized the library to reduce the differences among expressed genes to less than 10-fold among rare and abundant mRNAs [44,45]. Using this technique we were able to reduce redundancy in annotated genes from 33% in the non-normalized library to 11% after normalization. Second, we targeted specific sequences for subtraction: annotated cDNAs with high frequencies were targeted in order to focus effort on picking new, rare sequences. Through subtraction we were able to increase the rate of discovery of new annotatable sequences from 24% to 36%. However, analysis of these results indicate that a set of highly expressed sequences, some of which were not subtracted because they were not in the set of annotated genes, still make up much of the EST library and should be the focus of future subtractions.

### Gene annotation

**Figure 3:**
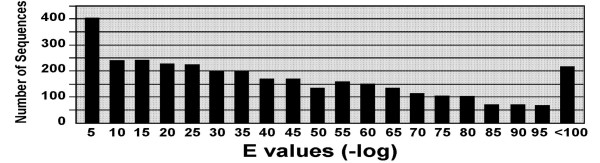
Distribution of e-values for annotations. Gene annotation is based on sequence similarity. The e-values, which describe the probability of random sequence similarity, are shown as the negative log value (e.g., 10^-5 ^= 5).

Of the 12,776 unique ESTs (3,776 clusters and 8,991 singletons), 3,877 (30%) were annotated. The distribution of e-values for these annotations is shown in Figure 3. Most (84%) of these ESTs have e-values less than 10^-5^. Among the clusters, 2,265 of 3, 779 (60%) were annotated as compared to 333 of 1,131 (30%) of confirmed high quality singletons. The lower percent of annotated singletons suggests that these are either rare fish-specific genes, or represent otherwise divergent, likely non-coding (5' or 3' UTR), regions.

Annotations for ESTs are based on similarity using BLASTX or BLASTN [42] to sequences in one of six-public databases: Swiss-Prot, Human UniGene, *Danio rerio *UniGene, *Oncorhynchus mykiss *Unigene, *Oryzias latipes *Unigene, or GenBank NR. *FunnyBase *includes locally parsed copies of these databases in a relational format. Thus, all searches are done locally and annotation features beyond the FASTA description can be queried. Consensus sequences from the *Fundulus *EST clusters as well as high quality single unique sequences (singletons) are used as query sequences for BLAST searches against these public databases. The use of consensus sequences, when available, allows sequences that do not contain regions of significant similarity with known protein (e.g., 5' or 3' noncoding regions) to be annotated if they are members of an annotated cluster. All hits with e-value less than 0.001 and their associated alignments are stored in the database and tracked with any associated functional annotation. Users can specify a custom level of significance when assessing the validity of homology based annotation. This record, which goes beyond storing a certain number of 'best hits', is critical because in many cases additional results may have a negligibly lower alignment score, but provide much more useful functional data.

**Figure 4:**
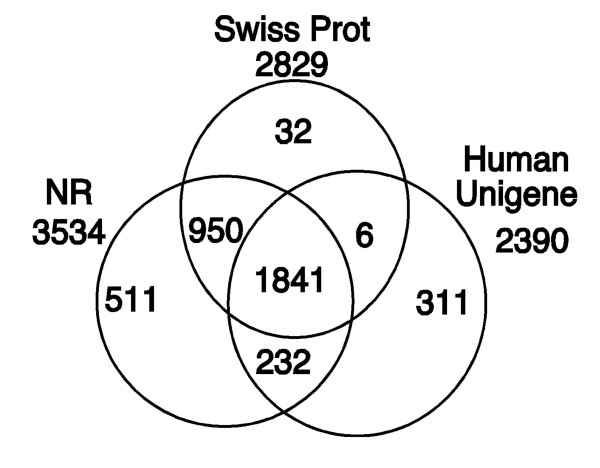
Venn diagram of annotations from three different databases. Number of unique ESTs annotated by three different databases: GenBank non-redundant (NR, total annotated = 3,534), Swiss-Prot (total annotated = 2,829), and human Unigene (total annotated = 2,390).

The use of multiple databases increases the total number of annotated ESTs (Fig. 4) as compared with any one source and provides opportunity to compare annotation between all three sources for 1,841 (47%) sequences. GenBank NR provided the most number of annotations, but these tend to be less informative (see *systematic functional annotations *below). Human Unigene provided an additional 311 (8% of total) annotations. SwissProt provided an additional 32 annotations (1% of total) with 743 fewer annotated sequences than the NR. However, SwissProt is uniformly well annotated as compared to NR where informative functional annotation can easily be buried by numerous uninformative hits at similar e-values. Besides increasing the number of annotations, comparing the annotations from multiple databases ensures that mistakes in the curation can be detected and information such as alternative gene names can be compiled from multiple sources.

### Systematic functional annotation: KEGG and Gene Ontology

In conjunction with performing similarity searches by BLAST, *FunnyBase *includes locally parsed representations of public databases such as SWISS-PROT in a relational database format. These databases provide additional information that cross-references other public resources such as GO, KEGG or OMIM [46] that are not available in the single FASTA description line returned by BLAST search.

KEGG [39] is a unique tool that represents metabolic and signal-transduction pathways both visually and computationally. *FunnyBase *links annotated genes to enzymes in KEGG pathways based on enzyme commission (EC) numbers. These pathway associations are stored and queries can readily identify genes from a given pathway that show specific patterns of expression. For visual inspection of the pathway, the web interface  links directly to the graphical KEGG pathways in which a gene occurs.

Of the 3,877 annotated ESTs in *FunnyBase*, 588 (14%) participate in one or more pathways defined by KEGG. These 588 ESTs represent 105 different pathways. Table 2 provides a breakdown of the number of ESTs in *FunnyBase *for the 10 pathways associated with the largest number of distinct sequences (contigs or singletons). The extent that a given pathway is represented in *FunnyBase *can be used to identify metabolic differences among tissues [47] or in different species.

**Table 2:** Number of distinct sequences in the Top 10 most common KEGG pathways. The KEGG pathway name and number of distinct sequences (clusters or singletons) from *FunnyBase *are presented. There are more distinct sequences than enzymes in a pathway because many enzymes have several protein subunits and many proteins have several different loci encoding the same subunit (e.g., NADH dehydrogenase, a protein complex of oxidative phosphorylation, has 26 protein subunits and 42 loci for these subunits).

**Sequence count for TOP 10 pathways**	
Glycolysis/Gluconeogenesis	89
Oxidative phosphorylation	86
Fatty acid metabolism	70
Pyruvate metabolism	69
Tryptophan metabolism	66
Butanoate metabolism	48
Glycerolipid metabolism	47
Valine, leucine and isoleucine degradation	46
Glycine, serine and threonine metabolism	44
Propanoate metabolism	44

The Gene Ontology project (GO) has produced a structured vocabulary in the form of an acyclic directed graph that biologists can use to annotate genes in a systematic manner [38]. *FunnyBase *includes two non-trivial steps to make the best possible use of GO terms. First, many GO annotations are lost if only the single 'best hit' from a homology search is considered because GO annotation is applied most often to a few model species such as human that may not appear as the single 'best hit' in a list of BLAST results. *FunnyBase *identifies the gene name associated with the 'best hit' BLAST result and then uses all GO annotation associated with hits from the complete BLAST results that have the same gene name as the 'best hit' and an e-value of e < 10^-12^. The goal of this approach is to identify annotation associated with a single 'best hit' gene based on results that may come from multiple species (orthologous genes) and therefore may have varying degrees of sequence similarity due to phylogenetic distance, but to avoid the problem of selecting an inconsistent set of GO terms arising from gene families that share regions of sequence similarity but may have different functions.

Secondly, GO annotation in public databases tends to annotate sequences with only the most specific GO term available, for example *RNA polymerase II transcription factor activity*, *enhancer binding *(GO:0003705) rather than the more general parent term *transcription regulator activity *(GO:0030528). However, in functional genomic analysis, significant patterns of expression may exist at the more general level of functional description. *FunnyBase *takes advantage of the connected parent-child relationship of GO terms provided by using the relational database version of GO available for download at  to identify such relationships. These data are used to extract the tree of more general GO terms related to those provided by public databases. A *FunnyBase *script then re-annotates genes with this more complete set of GO terms.

Of the 3,877 annotated genes, 1,912 (54%) are assigned one or more GO terms with a total 6,728 GO assignments being made directly based on information in public databases such as SwissProt. Using parent-child GO term relationship backtracking, an additional 34,112 GO term assignments were made, resulting in a final count of GO assignments of 36,024 excluding the most general terms that divide GO into three categories. Thus, on average, 19 GO terms are assigned to each of 1,912 annotated genes.

### Gene scaffolding: clustering of clusters

Humans have approximately 30,000 expressed genes, yet there are over 1,000,000 human UniGenes (NCBI). Clearly, these clusters of cDNAs greatly overestimate the number of unique genes. Similarly, FunnyBase has multiple clusters for the same gene: 15 apolipoprotein I, 10 cytochrome oxidase I, and 53 vitellogenin clusters. To provide a more precise estimate of the number of unique genes, consensus sequences were queried against the 27,695 sequences from the Human RefSeq [48] database, then grouped by identical gene symbol. Of the 2,376 *Fundulus *clusters that were similar to a sequence in Human RefSeq (e-value < 10^-10^), 1,818 (76%) had distinct gene annotations. This method of clustering clusters by similarity to well-annotated reference sequences provides a method to more accurately define the number of unique genes represented by an EST set.

### Case study: using functional annotation for microarray analysis

**Figure 5:**
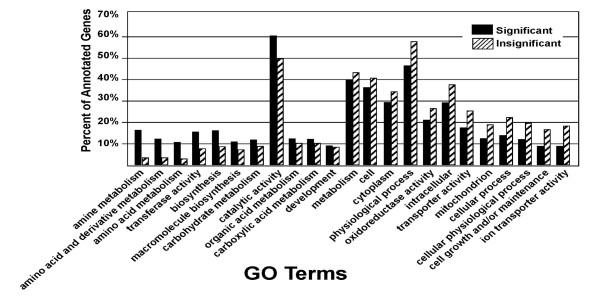
Distribution of significant and non-significant genes relative to GO terms. The relationship between gene expression from "common gardened" fish and the environment they evolved in was statistically analyzed and grouped by GO terms. Black bars represent genes whose levels of expression has a significant regression with the environmental thermal cline among populations (p < 0.05). Hatched bars represent the set of genes with no significant relationship to the thermal environment.

As a case study in how functional annotation in *FunnyBase *can be integrated with microarray data in a rigorous manner, we used a data set based on a microarray of metabolic genes printed from ESTs annotated in *FunnyBase *[47]. Statistical analysis of this set of 363 metabolic genes identified a set of 62 genes that showed statistically significant regression between gene expression levels and temperature along the Atlantic coast. That is, among individuals collected from different locations along the thermocline and then acclimated to common physiological conditions for at least nine months before analysis, 17% of the metabolic genes had a linear relationship between the amount of mRNA and the environmental temperature these animals evolved in. Our hypothesis was that this set of 59 genes represents a functionally different set than those genes that do not show regression with temperature. To test this hypothesis we examined the frequency of genes annotated with a given GO term in the statistically significant gene set versus the non-significant genes. Figure 5 shows the relevant proportions in each set for GO terms that are represented by 5 or more ESTs in the significant set. For example, the GO term *Amine Metabolism *(GO:0009308) is assigned to 14% of the 62 statistically significant genes but only 3% of the non-significant genes (those that do not show significant regression with temperature). A Fisher-exact test indicates these frequencies (14% vs. 3%) represent different underlying distributions (p < 0.001). Specifically, genes involved in amine metabolism are overrepresented in the set of genes that show regression with temperature as compared with the remaining sequences. This significant increase is found for two other non-mutually exclusive GO terms: *amino acid and derivative metabolism*, and *amino acid metabolism *(p < 0.05). Other GO terms show a reverse trend although none were statistically significant. For example, ion transport (p = 0.08) and cell growth (p = 0.16) had few genes with a clinal variation in expression. These data suggest that the functions of genes influence whether they are affected by ecologically interesting patterns of expression (Fig 5).

### Web interface

The web interface  provides public access to the *FunnyBase *system and dataset. Searches can query by keyword in annotation, gene name, GO term, metabolic pathway, clone or plate id, and BLAST homology search. All data including raw sequences, cluster memberships, cluster alignments, and alignments with homologous sequences are provided for the user to examine the source of annotations. Links associated with each annotation are made to external resources such as GO's AMIGO browser, KEGG pathways, SwissProt, and NCBI records.

### Other Fundulus sequences

*FunnyBase *was constructed to annotate sequences for the analysis of gene expression. It provides identification and annotation for genes in the Crawford laboratory with a primary goal of identifying clones useful for the construction of microarrays. As such, other *Fundulus *sequences in Genbank are not included. However, *FunnyBase *forms the basis of the TIGR Killifish gene index  that includes publicly available *F. heteroclitus *sequences.

## Conclusions

Customized species specific EST databases are available for many species [17,18,21,49-56]. *FunnyBase *provides an integrated method to annotate ESTs with the most biologically relevant set of associations and provides several innovations for the production of ESTs for microarrays. Control sequences are identified in each 96-well plate so that mislabelled or inverted plates are automatically detected. Annotations are based upon several different public databases. The multiple annotations provide greater assurance about gene description and greater frequency of annotation than any one database. The most functionally informative innovation of *FunnyBase *is the process of culling through numerous primary similarity search results in order to identify links to systematic functional databases in GO and KEGG. These provide a discrete set of terms that can be analyzed statistically and that are organized into networks that represent biological knowledge of higher-level functional and pathway associations. The range of databases queried by similarity search and the tracking of homology beyond a single 'best' hit maximizes the opportunity to obtain this annotation. A richer set of GO terms is achieved by using all hits with e-values less than 10^-11 ^that represent the same gene as the 'best hit'. Additional GO terms that represent more general functions than those found in public annotation are derived through the parent-child relationship of the Gene Ontology. EC numbers provide links, *via *KEGG, to metabolic pathways and these stored terms can be used to investigate the relationship between gene expression in specific metabolic pathways including cardiac metabolism [57]. To provide a more accurate accounting of the number of unique genes, consensus sequence from clusters of ESTs were queried against the Human RefSeq database and those sequences sharing the same gene symbol are grouped based on this scaffolding information. These approaches use publicly available bioinformatics tools (BLAST, CAP3, Phred, Cross-Match, Perl, and the MySQL database management system). The application of theses tools in an appropriate framework as outlined in *FunnyBase *can be used to create a systems level functional genomics annotation system useful for EST databases to study biological processes among a rich diversity of organisms.

## Methods

### Organism

The animal protocols used in the present study have been approved by the University of Miami Institute Animal Care and Use Committee. The teleost fish *Fundulus heteroclitus *used for ESTs were collected from two sites: Scorton Creek in Sandwich, MA, and Stone Harbor, NJ. These populations are in the central portion of the thermal cline and have relatively high levels of heterozygosity [32]. These fish were subjected to the following environmental regime before tissues were harvested for mRNA extraction: kept in controlled temperature and aeration conditions, and acclimated to common conditions (20°C, 15 ppt salinity) in re-circulating aquaria for at least nine months before experiments. Following this common acclimation a subset of fish were subjected to one of several stresses: 4°C, 34°C, hypoxia, or a complex mix of hydrocarbons.

### cDNA library

To effectively isolate and sequence thousands of cDNAs for the production of microarrays, a unidirectional cDNA library with few non-recombinants was required. We created four cDNA libraries: heart libraries from non-stressed and stressed fish and liver libraries from non-stressed and stressed fish. The non-stressed *F. heteroclitus *cardiac and liver libraries were provided by Drs. S. Karchner and M. Hahn, WHOI [58] and were constructed using the UniZap λ cDNA Gigpack Gold cloning kit (Stratagene, La Jolla, CA, USA). The cardiac library was produced from 27 fish hearts (both sexes) sampled from Scorton Creek in Sandwich, MA. The cDNAs in these libraries are oriented such that the 5'end of each cDNA is ligated to EcoR1 and 3' poly A is ligated to XhoI. These libraries had less than 1% non-recombinants, i.e. 2 of 300 random clones from a non-normalized library had no inserts. The stressed libraries included 4 fish subjected to the four stressors (above) and 4 non-stressed individuals. Unidirectional heart and liver libraries were constructed such that the 5'end of each cDNA is ligated to EcoR1 and 3' poly A is ligated to XhoI of the plasmid vector pSmart (Lucigen, Middleton, WI, USA). The pSmart-cDNA vector was designed for EST work. The vector expresses kanamycin-resistance and has a terminator on both sides of the cDNA insertion site preventing expression of cDNA. These two attributes (non-expression and Kan-resistance) increase the stability of different genes in the library versus cDNA libraries in Amp libraries with Lac promoters (Crawford, unpublished). These libraries had less than 1% non-recombinants.

Normalization of cDNA libraries reduces the differences among expressed genes to less than 10-fold among rare and abundant mRNAs [44,45]. Normalized libraries were produced by isolating cDNAs from approximately 10^12 ^plasmids. The cDNAs were isolated using PCR amplification with vector specific primers immediately 5' and 3' to the insertion site (EcoRI and XhoI sites). These PCR products (PCR-cDNAs) were denatured and hybridized to single stranded plasmids from the cardiac cDNA library. Taking advantage of Cot values, the most abundant cDNAs were annealed to the more abundant PCR products and were removed selectively by hydroxyapatite-column chromatography. The single-stranded plasmids in the flow-through were converted to double strands using the Sequenase DNA polymerase (Amersham, Piscataway, NJ, USA). DH10s *E. coli *(BRL) were transformed with these double-stranded plasmids by electroporation. The number of recovered plasmids and the resulting complexity of the normalized library depended on the duration of hybridization or Cot values. Two normalized libraries were made using either a 12 or 24 hour hybridization. The library from the 12-hour hybridization yielded 250,000 plasmids. The library from the 24 hour hybridization yielded 3,000 plasmids and had a greater representation of rare mRNAs and greater frequency of non-recombinants.

### Isolation and sequencing of cDNAs

Characterization of cDNAs (growth of individual bacterial colonies containing plasmids, PCRs, purification of PCR products, sequencing reactions) used 96 well plates and octopipettes. To characterize cDNAs, 96 individual bacterial colonies from the normalized library were randomly chosen, and each was grown in 1.25 ml of Superbroth in 2 ml-96 well plates. After 18 hours of growth, two 250 ul bacterial glycerol stocks were made and stored in 96 well plates at -80°C. One microliter of these bacterial growths was used for PCR reactions using forward and reverse plasmid specific primers: (PucF = CGCCAGGGTTTTCCCAGTCACG, PucR = GAGCGGATAACAATTTCACACAGGAAA). PCR reactions had 0.2 mM dNTPs, 10 pmoles of each primer, 1 unit of Promega Taq (0.2 ul), and reaction buffer with detergents and DMSO (final concentrations: 50 mM Tris HCl, pH 9.2 (25°C), 16 mM (NH_4_)2SO_4_, 2.25 mM MgCl_2_, 2% (v/v) DMSO, 0.1% (v/v) Tween 20). Two-step thermal cycle conditions were used (94°C for 10 seconds; then 32 cycles of 94°C for 30 seconds followed by 70°C for 5 minutes; then 72°C for 15 minutes). PCR products were purified manually in 96 well format using Sephadex G-50 in a deep well plate with a 0.2 microfilter (Millipore, Billerica, USA) or robotically using AmPure (Agencourt, Beverly, MA, USA) and EvolP^3 ^96 pipetting liquid handling system (PerkinElmer Life Sciences Inc., Boston, MA, USA).

PCR products were sequenced from the 5' end (relative to the mRNA) on an ABI 373 or ABI 3730 sequencer using ABI "Big Dye" reaction mix. We typically used 1/16 the amount of reaction mix, yielding 300 to 400 unambiguous bases. Sequences were purified using biotin primers and streptavidin coated magnetic beads (for the ABI 373) or Agencort CleanSeq (for the ABI 3730).

### Validation

We used three procedures to verify that the correct sequence was associated with each cDNA. 1) Each 96-well plate had three wells with a "marker cDNA" (*Ctenophore *cDNA #5, a random cDNA with no similarity to any sequence in GenBank). Two wells (#40 and #67) always contained the marker cDNA, and thus any misloading or mislabeling of sequencing lanes was identifiable. The third marker cDNA was placed in a well that corresponds to the plate number (e.g., plate 2 had the marker in well 2). 2) After the production of 12 plates, one row (8 wells) from each plate was re-sequenced. Thus, 8/96 or ~8% of all sequences and their locations were confirmed. 3) cDNAs used for microarrays were re-sequenced. These measures are important to ensure that the correct and known cDNAs are printed.

### Subtraction

The complexity of the normalized library was reduced by subtracting the characterized cDNAs previously isolated from the normalized library. Subtraction greatly reduced the probability of isolating the same cDNA and thus improved the efficiency of screening the library for unique clones. Subtraction used a 100-fold molar excess of biotin-labeled antisense cDNAs produced by PCR using all the characterized cDNAs as substrates and vector-specific primers in which the 3' primer was labeled with biotin. These PCR products were hybridized to the cDNA libraries in the presence of oligo-dA and vector-specific oligos (that prevented non-specific hybridization to oligo-dT or vector sequences). After a 24 hour hybridization, genes in the library that bound to these biotin-labeled PCR products were removed with the use of magnetized, streptavidin coated beads. DH10s *E. coli *were transformed with the subtracted library by electroporation.

### Hardware and software

Computational work was done on an Apple G5 dual 2 GHz processor system with 4 GB of RAM. Data are stored in a MySQL database, perl scripts were used extensively for parsing and loading data, and PHP was used on an APACHE web server to construct the user interface. Additional programs available from their authors are mentioned within context. Software and databases are described in Table 3.

**Table 3:** Software and Databases. Publicly available software and databases used for *FunnyBase*. The version and/or download date are listed.

**Resource**	**Version and/or Download Date**
**Software**	
Stand-alone BLAST	2.2.8
Cross match	0.990329
CAP3	January, 2004

**Public Sequence Similarity Databases**	
Swiss-Prot	
NR	44
Human RefSeq	June, 2004
Human Unigene	June, 2004
Zebrafish Unigene	June, 2004
Medaka Unigene	June, 2004
Rainbow Trout Unigene	June, 2004
	June, 2004

**Functional Annotation**	
Gene Ontology	2004-06-04
KEGG	June, 2004

### Microarrays

Microarrays were printed using a select 384 cDNAs from *F. heteroclitus *cardiac library encoding essential proteins for cellular metabolism isolated from over 40,000 expressed sequences . These 384 cDNAs were amplified with amine-linked primers and printed on 3-D Link Activated slides (Surmodics Inc., Eden Prairie, MN, USA) using *GeneMachine OminGrider*, and blocked following slide manufacturer protocols. The suite of 384 amplified cDNAs was printed as a group in four spatially separated replicates. Four hybridization zones of these four replicate arrays were printed per slide, with each zone set separated by a hydrophobic barrier. Samples were hybridized twice; once with Cy3 and once with Cy5 resulting in overall technical replication of 8-fold per sample.

### Sample preparation and hybridization

RNA was extracted from tissue homogenate in a chaotropic buffer using phenol/cholorform/isoamyl alcohol and RNA quality was assessed using the Agilent 2100 Bioanalyzer (Agilent Technologies, Palo Alto, CA, USA). RNA for hybridization was prepared by amplification using a modified Eberwine protocol [59] using the Ambion Amino Allyl MessageAmp aRNA Kit. Cy3 and Cy5 were hybridized to slides and incubated 12–18 hours at 42°C. Following hybridization, slides were scanned using the Packard Bioscience ScanArray Express microarray scanner (PerkinElmer Life Sciences Inc., Boston, MA, USA) and images processed using ImaGene (Biodiscovery Inc., Marina del Rey, CA, USA).

## Authors' contributions

JP designed, scripted and implemented *FunnyBase *and provided statistical analyses of database. MFO initiated, designed protocols and provided sequences for *F. heteroclitus*' EST project. JDV optimized robotic interfaces for sequencing and sequenced ESTs. JLR and KJK sequenced ESTs. GJW collaborated on bioinformatics and database development. JAW provided microarray data and analyses. DLC initiated *F. heteroclitus*' EST project and developed the database and annotation schemes for *FunnyBase*. All authors read and approved the final manuscript.
